# Real-time temperature anomaly detection in vaccine refrigeration systems using deep learning on a resource-constrained microcontroller

**DOI:** 10.3389/frai.2024.1429602

**Published:** 2024-08-01

**Authors:** Mokhtar Harrabi, Abdelaziz Hamdi, Bouraoui Ouni, Jamel Bel Hadj Tahar

**Affiliations:** ^1^Department of Computer Engineering ISITCOM, University of Sousse, Sousse, Tunisia; ^2^NOOCCS Research Lab, ENISO University of Sousse, Sousse, Tunisia

**Keywords:** deep learning, convolutional auto encoder, anomaly detection, real-time monitoring, refrigeration systems, vaccine

## Abstract

Maintaining consistent and accurate temperature is critical for the safe and effective storage of vaccines. Traditional monitoring methods often lack real-time capabilities and may not be sensitive enough to detect subtle anomalies. This paper presents a novel deep learning-based system for real-time temperature fault detection in refrigeration systems used for vaccine storage. Our system utilizes a semi-supervised Convolutional Autoencoder (CAE) model deployed on a resource-constrained ESP32 microcontroller. The CAE is trained on real-world temperature sensor data to capture temporal patterns and reconstruct normal temperature profiles. Deviations from the reconstructed profiles are flagged as potential anomalies, enabling real-time fault detection. Evaluation using real-time data demonstrates an impressive 92% accuracy in identifying temperature faults. The system’s low energy consumption (0.05 watts) and memory usage (1.2 MB) make it suitable for deployment in resource-constrained environments. This work paves the way for improved monitoring and fault detection in refrigeration systems, ultimately contributing to the reliable storage of life-saving vaccines.

## 1 Introduction

Ensuring the efficacy of vaccines hinges on maintaining consistent and accurate temperatures within refrigeration systems. However, malfunctioning temperature sensors can have devastating consequences, leading to vaccine spoilage, safety hazards, and significant resource waste (Trump et al., 2022; Viswanath et al., 2022; Wanyonyi et al., 2024). Anomaly detection plays a pivotal role in this process by identifying deviations from expected temperature patterns, which can indicate potential issues such as sensor faults or environmental fluctuations. It’s important to note that while our system primarily focuses on anomaly detection, the anomalies detected may encompass a range of deviations, including those stemming from sensor reading faults. The distinction between anomaly detection and fault detection is essential to understand: anomaly detection involves identifying any deviation from the expected behavior, while fault detection specifically targets the identification of malfunctions or errors within the sensor readings themselves. In our context, anomalies detected by our system may include those resulting from sensor reading faults, underscoring the interconnected nature of anomaly detection and fault detection.

**Figure 1:**
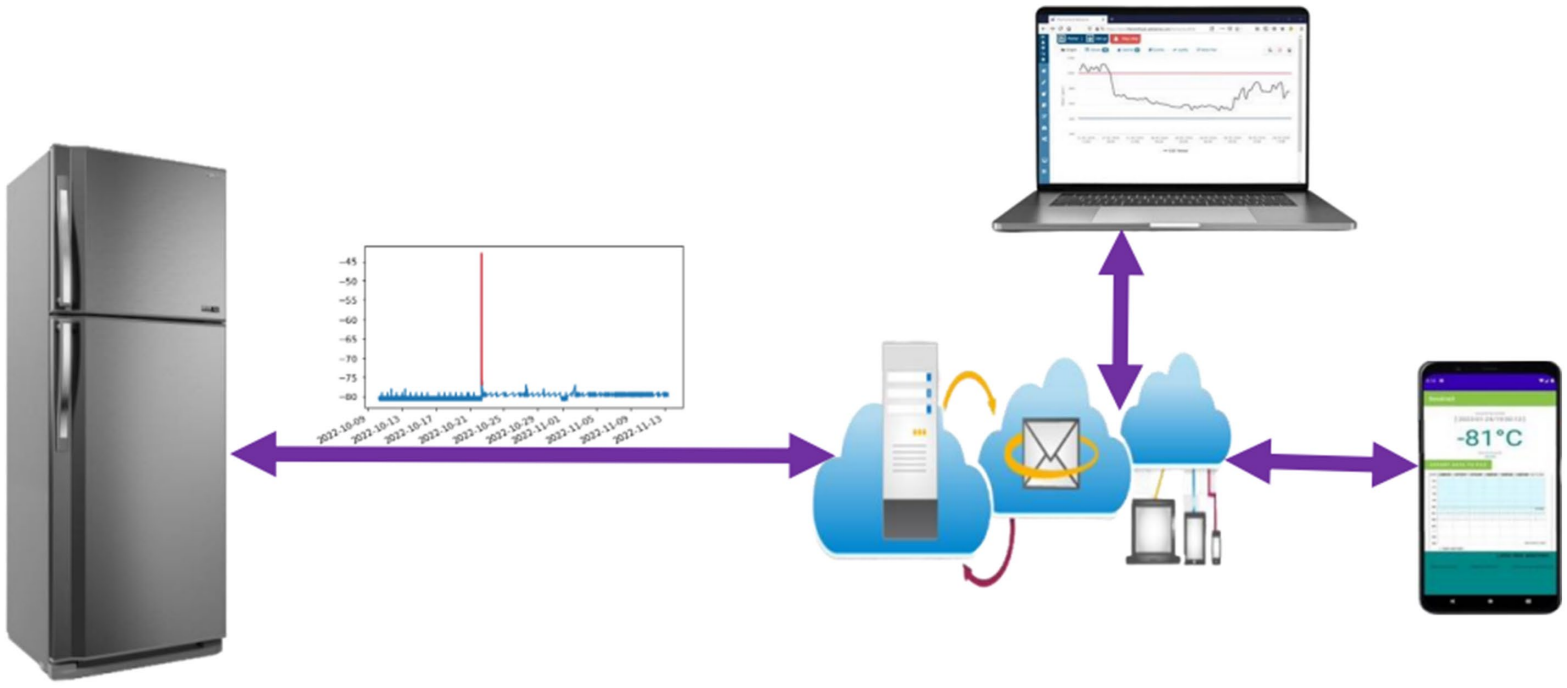
Designed system.

This paper proposes a comprehensive Internet of Things (IoT)-based solution for real-time temperature monitoring of vaccine storage refrigeration systems. As depicted in Figure 1, the system leverages a novel deep learning model on an ESP32 microcontroller to analyze sensor data and detect anomalies or potential sensor faults. Users can remotely monitor the temperature and receive alerts about these issues through a mobile application with an internet connection, enabling early intervention and ensuring the safe storage of vaccines. This approach combines the power of deep learning for anomaly detection with the convenience of remote monitoring via a mobile application, providing a comprehensive and efficient solution for real-time temperature management in vaccine storage systems. Traditional methods, like threshold-based approaches, are simple to implement but lack sensitivity to subtle anomalies and can trigger false alarms due to environmental fluctuations (Corradino et al., 2023; Jeffrey et al., 2023; Liu et al., 2023; Harrou et al., 2024). Statistical methods offer more robustness but struggle to capture early signs of sensor degradation or subtle temperature drifts (Harrou et al., 2023; Kartal, 2023; Zou et al., 2023). Both approaches rely on assumptions about normal temperature distribution, which may not always hold true in real-world scenarios. Recent advancements in machine learning offer promising alternatives.

While Artificial Neural Networks (ANNs) and Support Vector Machines (SVMs) can learn sensor behavior, they can be computationally expensive (El-Shafeiy et al., 2023; Kurani et al., 2023). Recurrent Neural Networks (RNNs), particularly Long Short-Term Memory (LSTM) networks, excel at handling time-series data like temperature readings due to their ability to capture temporal dependencies, making them effective in anomaly detection for sensor faults (Yin et al., 2020; Farahani, 2021). While LSTMs hold promise for real-time temperature fault detection, their computational demands can be a hurdle for deployment in resource-constrained environments with low-power microcontrollers, commonly found in vaccine storage refrigeration systems (Badidi et al., 2023; Himeur et al., 2023; Kumar and Agrawal, 2023). This limitation necessitates exploring alternative deep learning approaches that offer a balance between anomaly detection efficacy and computational efficiency.

A thorough review of existing literature reveals a gap in research regarding the application of autoencoders for temperature fault detection in refrigeration systems deployed on microcontrollers. While several studies explore fault detection using machine learning for temperature data, let us compare them to our work with a focus on resource-constrained environments:

Work 1 as discussed in Lemes et al. (2023). Explores anomaly detection in refrigeration systems using Wavelet Transform. While demonstrating an efficient approach, it relies on a specific mathematical transformation and may not directly translate to a machine learning model suitable for deployment on microcontrollers.

Work 2 by Soltani et al. (2022). Investigates the use of various machine learning algorithms like SVM and CNN for fault diagnosis in refrigeration systems. While effective, their work often relies on simulated data or computational models which may not capture the complexities of real-world scenarios. Additionally, these algorithms can be computationally expensive for deployment on resource-constrained devices.

Other studies explore functionalities beyond core temperature fault detection:

Work 3 by Liu et al. (2022). Focuses on real-time temperature prediction in data centers using a state-space model. While valuable for temperature control, it does not directly address fault detection.

Work 4 by Fadaeefath Abadi et al. (2022). Emphasizes the significance of fault detection for system reliability in vapor compression refrigeration systems. Their work complements ours by highlighting the importance of the problem we address.

Research on smart refrigerators has explored various applications of the Internet of Things (IoT) for functionalities beyond temperature control:

Work 5 by Mohammad et al. (2020). Proposes a smart refrigerator system using a Convolutional Neural Network (CNN) to detect food items and manage inventory. While showcasing the potential of IoT, their work focuses on a different aspect of refrigerator management compared to our core objective of temperature fault detection.

Work 6 by Nasir et al. (2018). Describes an IoT-based smart refrigerator system that utilizes sensors to monitor food spoilage and alert users. Similar to Work 5, this research tackles a separate functionality within smart refrigerators.

Harrabi et al. (2023) present a valuable review of existing literature on IoT-based solutions for monitoring the storage of vaccines and other sensitive materials.

### 1.1 Our distinction

This work focuses on anomaly detection in temperature sensor readings, which can be a precursor to potential faults. Faults represent complete failures of the sensor, rendering it inoperable. In contrast, anomalies signify deviations from the expected sensor behavior, indicating potential issues before they escalate into complete failures. By identifying anomalies through reconstruction error analysis in our CAE model, we can take preventive measures to address potential problems before they occur, ensuring the safe storage of vaccines.

### 1.2 This paper’s contribution

This paper addresses the limitations of existing monitoring systems by proposing a novel deep learning-based solution integrated within a remote monitoring system for real-time temperature sensor anomaly and fault detection in refrigeration systems used for vaccine storage. The system leverages a CAE model trained on real-world data, enabling:

Early detection of deviations: the CAE model facilitates anomaly detection through reconstruction error analysis. This allows the system to identify potential problems, such as sensor malfunctions or gradual drifts in temperature readings, before they escalate into complete sensor failures.

Real-world data training: as highlighted before, training on real data ensures the model learns the nuances of sensor behavior in a real refrigeration system, allowing it to differentiate between normal variations and anomalies that might indicate underlying issues.

Resource-constrained deployment: optimization for the ESP32 microcontroller facilitates practical implementation within the constraints of real-world IoT applications.

Continuous monitoring: the ESP32 enables continuous monitoring of temperature readings and other critical parameters within the refrigeration system.

Wireless data transmission: the ESP32 can be programmed to transmit data wirelessly for remote monitoring.

Alert triggers: the system can trigger alerts in case of sensor faults, electrical cutoffs, or prolonged door openings.

By incorporating anomaly detection alongside fault detection, this paper strives to contribute a practical and efficient deep learning solution for real-time temperature sensor monitoring within a remote monitoring system for vaccine storage refrigeration systems. This approach allows for early intervention, potentially preventing complete sensor failures and ensuring the safe storage of vaccines.

The following sections will delve deeper into the methodology behind our CAE model, detailing its architecture and training process. We will then present the experimental setup and results, showcasing the effectiveness of our approach in real-world scenarios. The discussion section will explore the implications of our findings and potential avenues for future research, culminating in a comprehensive understanding of the proposed solution’s significance.

## 2 Background

Maintaining consistent and accurate temperatures within refrigeration systems is crucial for effective vaccine storage. Even minor deviations from the optimal temperature range can compromise vaccine efficacy and safety. Anomaly detection plays a vital role in this process by identifying sensor faults that can lead to such deviations. This section explores various anomaly detection methods commonly used for sensor fault detection in refrigeration systems.

### 2.1 Statistical methods

Statistical methods have traditionally served as the foundation for anomaly detection due to their simplicity and interpretability (Chalapathy and Chawla, 2019; Barbariol et al., 2022). However, their effectiveness can be limited in complex scenarios:

Threshold-based detection: this straightforward method sets predefined upper (T_max_) and lower (T_min_) temperature thresholds. Any reading exceeding these thresholds triggers an anomaly alert. However, this approach lacks sensitivity to subtle anomalies and is prone to false alarms due to environmental fluctuations (Patil et al., 2022).

Z_score_ (z): This method utilizes historical temperature readings to calculate the standard deviation (σ) and mean (μ). For each new reading (T_new_), the (Z_score_)is calculated as:


(1) $$Z=\frac{T_{\mathrm{new}}-\mu }{\sigma }$$

An anomaly is flagged if the absolute Z-score exceeds a predefined threshold. While more robust to environmental variations, this method assumes a normal distribution of temperature data, which may not always hold true.

### 2.2 Distance-based methods

These methods compute a “distance” metric between data points and a reference model representing normal behavior, identifying anomalies based on predefined distance thresholds (Talagala et al., 2021; Agrawal, 2022; Liu et al., 2023).

Euclidean distance: a common metric, it calculates the straight-line distance between a new data point (T_new_) and a reference point (T_ref_) in n-dimensional space (representing temperature readings over time). The Euclidean distance is defined as:


(2) $$d=\sqrt{\overset{n}{\underset{i=1}{\sum }}{\left(Tnew\;\left[i\right]-Tref\;\left[i\right]\right)}^{2}}$$

where n is the number of temperature readings and d is the calculated distance. An anomaly is detected if the computed distance exceeds a predefined threshold.

### 2.3 Machine learning and deep learning techniques

AI-based methods offer diverse approaches that can capture complex patterns and relationships in data. Some prominent examples include:

Recurrent neural networks (RNNs): capture temporal dependencies in time-series data like temperature readings.

Variational autoencoders (VAEs): learn latent representations of data while incorporating a probabilistic element for anomaly detection (Liu et al., 2023).

Generative adversarial networks (GANs): train two models: a generator that learns to create realistic data, and a discriminator that tries to distinguish real data from generated data. This adversarial training can be used to identify anomalies that deviate from the learned data distribution (Choi et al., 2023; Wang et al., 2023).

Attention mechanisms: focus on specific parts of the input data that are most relevant for the task (e.g., focusing on specific time points in a temperature sequence; Kasgari et al., 2023).

Graph neural networks (GNNs): can be used if the data has a graph-like structure, where nodes represent sensors and edges represent relationships between them (Jiang and Luo, 2023).

Autoencoders: Autoencoder-Based Anomaly Detection for Imbalanced Data Industrial process monitoring often encounters imbalanced datasets, where anomaly labels represent a small fraction (5–10%) compared to normal data (Ji et al., 2023; Yang et al., 2023; Dissem et al., 2024). This imbalance poses a challenge for deep learning models due to their reliance on large amounts of positive data for effective training (Gao et al., 2024). Traditional techniques like dropout and batch normalization may not adequately address this issue (Saeedi and Giusti, 2023). Undersampling, a common approach for balancing datasets, can also lead to reduced accuracy (Troullinou et al., 2023).

The autoencoder consists of an encoder and decoder network. The encoder transforms high-dimensional input data into a lower-dimensional latent space, while the decoder reconstructs the original data from this encoded representation (Solera-Rico et al., 2024). In this work, a simple three-layer autoencoder architecture is employed, consisting of an input layer, a hidden layer, and an output layer.

The input to the autoencoder is denoted as a vector x_t_ ∈ ℝ^dx^, where dx represents the input dimensionality. The encoder maps this vector to a latent representation m_t_∈ ℝ^da^, and the decoder reconstructs an estimated version x_t_ of the original input using the following equations:


(3) $$m_{t}=\sigma_{1}\left(W_{1}x_{t}+b_{1}\right)$$

(4) $$x_{t}=\sigma_{2}\left(W_{2}m_{t}+b_{2}\right)$$

Here, σ_1_ and σ_2_ represent activation functions, and W_1_, W_2_, b_1_, and b_2_ denote the learnable parameters for the encoder and decoder, respectively.

To prevent overfitting, an L_2_ regularization term is incorporated into the cost function:


(5) $$J\left(W_{1},\;W_{2},\;b_{1},\;b_{2}\right)=N_{1}\overset{N}{\underset{t=1}{\sum }}∥\;x_{t}-{\hat{x}}_{t\;\;∥\;}22+\lambda \left(∥W_{1}∥\;22\;+W_{2}∥\;22\right)$$

where λ is the regularization coefficient, controlling the trade-off between training error and model generalizability.

Anomaly detection: following training, the reconstruction error vector e_t_= $x_{t}-{\hat{x}}_{t}$ is calculated for each data point. The root-mean-square error (RMS) of this vector serves as the anomaly score. An observation x_t_ is classified as anomalous if RMS(e_t_) > θ, where θ is a decision threshold optimized based on the true positives and false positives in the training dataset. By leveraging the autoencoder’s capability to learn normal data patterns and identify deviations, this approach aims to enhance fault detection and analysis in industrial processes with imbalanced data.

Autoencoders, particularly prominent in fault detection applications, learn to reconstruct the input data from a compressed representation. This ability allows them to effectively identify anomalies and deviations from expected patterns. Our paper focuses on exploring the application of autoencoders for temperature sensor fault detection in refrigeration systems, comparing their advantages, limitations, and performance against traditional statistical methods and other AI-based approaches.

In conclusion, this background section has explored the landscape of anomaly detection methods for sensor fault detection in refrigeration systems. While traditional statistical and distance-based techniques offer a foundation, their limitations become apparent when dealing with complex, real-world sensor data. Machine learning and deep learning approaches hold immense promise in capturing these intricate patterns. Among these, autoencoders emerge as a particularly compelling choice. Their ability to learn efficient representations of normal sensor data allows them to effectively identify deviations that signal potential anomalies. This, coupled with their moderate computational complexity, makes them highly suitable for deployment on resource-constrained devices like the ESP32 microcontroller within a remote monitoring system for vaccine storage refrigeration. By leveraging autoencoders, such a system can achieve accurate and timely anomaly detection, ensuring the optimal functioning of crucial refrigeration units for critical vaccine storage.

## 3 Materials and methods

This section details the hardware, software tools, and methodology employed to develop and implement the deep learning-based solution for real-time temperature sensor fault detection in refrigeration systems used for vaccine storage.

### 3.1 Materials

The study utilized a combination of hardware and software tools:

The anomaly detection system utilizes an ESP32 microcontroller for real-time data collection and model deployment, enabling on-device anomaly detection. The ESP32 is paired with a high-precision PT100 temperature sensor to gather accurate temperature readings within the refrigeration system. To build, train, and evaluate the time series model for anomaly detection, open-source deep learning frameworks TensorFlow and Keras are used.

### 3.2 Methods

The methodology involved a series of steps, encompassing data acquisition and preprocessing, model development, evaluation, and deployment on the ESP32 microcontroller.

#### 3.2.1 Data acquisition and pre-processing

##### 3.2.1.1 Data collection

Each sample or point in our dataset represents a single temperature reading taken every 5 min from the PT100 sensor. Over a 24-h period, this results in 288 samples (24 h * 60 min /5 min). To ensure that these samples are collected and processed in real-time, we utilize FreeRTOS as the operating system on our microcontroller. FreeRTOS allows us to manage concurrent threads with specified periodicity and priority, ensuring that the sampling task is performed precisely every 5 min without missing any deadlines. The time required for processing each sample is significantly shorter than the 5-min interval, allowing the microcontroller to enter sleep mode between readings, thereby conserving energy and enhancing the system’s efficiency.

By employing FreeRTOS, we ensure that our system respects real-time constraints while maintaining energy efficiency, crucial for continuous operation in remote or resource-constrained environments.

The data acquisition system was set up as follows:

Sensor: PT100, chosen for its high accuracy and stability in temperature measurement.

Microcontroller: ESP32, selected for its processing power and wireless communication capabilities.

Converter: MAX31865, used to convert the analog signal from the PT100 sensor to a digital format compatible with the ESP32.

The temperature readings were transmitted to a Firebase real-time database, which provided an efficient platform for data storage and potential remote monitoring. This setup facilitated continuous data logging and easy access for subsequent analysis.

##### 3.2.1.2 Data pre-processing

Pre-processing of the collected data is crucial to ensure its quality and suitability for training the Convolutional Autoencoder (CAE) model. The pre-processing steps included data cleaning, normalization, and splitting, as detailed below:

##### 3.2.1.3 Data cleaning

Inconsistency checks: the data was examined for any inconsistencies, such as abrupt spikes or drops that could indicate sensor malfunctions or transmission errors.

Outlier removal: in our data processing workflow, outliers are identified as temperature readings that fall outside the logical range due to issues such as sensor malfunctions, bad contacts, noise, or other problems. When an outlier is detected, the system sleeps for 2 s and retries the reading process up to 10 times (20 s total). If the outlier persists after 10 attempts, it is flagged as a potential sensor fault without the need for an AI model. Conversely, readings within the logical range are sent to the model for classification, where they are analyzed for potential anomalies based on historical patterns. This entire process, from reading to outlier detection and classification, does not exceed 21 s, making the 5-min interval sufficient for effective monitoring.

Error handling: missing or invalid readings were addressed by using interpolation methods to estimate reasonable values based on neighboring data points.

###### 3.2.1.3.1 Normalization

Scaling: to ensure all data points contribute equally during model training, the temperature readings were normalized to a specific range, typically between 0 and 1. This scaling helps in preventing features with larger scales from dominating the learning process and improves the convergence rate of the model.

Data splitting: we generated two separate datasets to represent normal and abnormal behaviors, each containing 8,640 data points. These datasets were then combined and split into training, validation, and testing sets.

Training set: the combined dataset was divided into three subsets: training, validation, and testing. The training set comprised 70% of the total data (12,096 data points), including 6,048 normal and 6,048 abnormal readings. This ensured that the autoencoder learned to reconstruct “normal” data patterns without being influenced by anomalies.

Validation set: a smaller subset of the data (15%, or 2,592 data points), including 1,296 normal and 1,296 abnormal readings, was set aside as the validation set. This set was used throughout the training process to fine-tune hyperparameters and monitor the model’s performance on unseen data, helping to avoid overfitting.

Testing set: the remaining 15% of the data (2,592 data points), including 1,296 normal and 1,296 abnormal readings, was used as the testing set. This set served to evaluate the autoencoder’s ability to differentiate between normal and anomalous data by observing the reconstruction errors.

##### 3.2.1.4 Visualization and initial analysis

Before training the CAE model, it was essential to understand the characteristics of the dataset through visualization. This step provided insights into the overall stability of the temperature readings and highlighted instances of anomalies.

**Figure 2:**
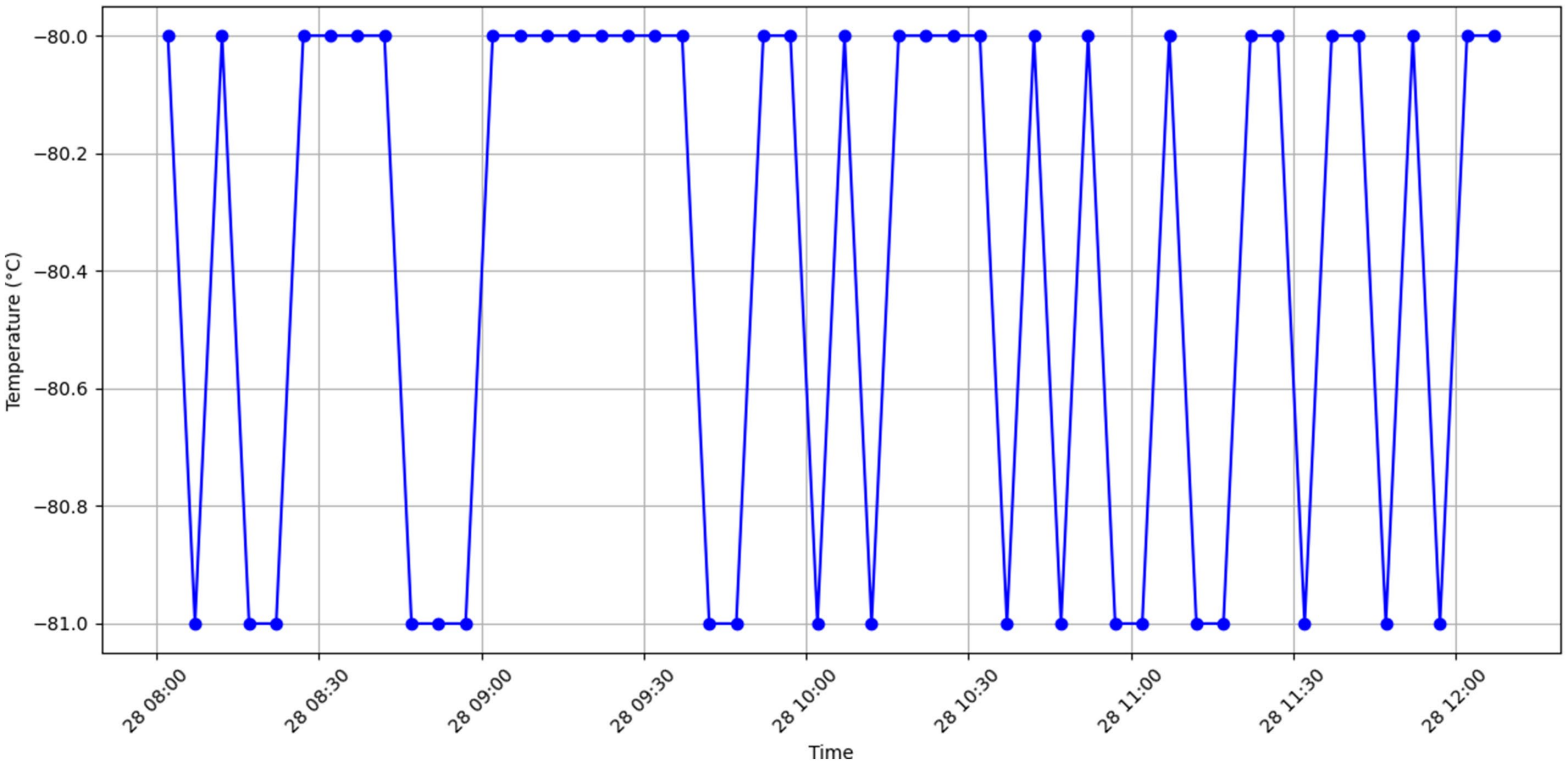
Stable and expected temperature behavior.

Normal data visualization: Figure 2 displays a sample of normal temperature readings over a day, illustrating the expected stability and periodicity of the data.

**Figure 3:**
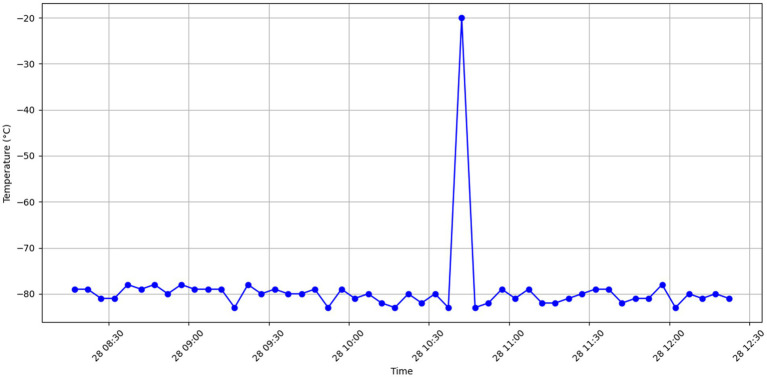
Anomalous temperature fluctuations.

Anomalous data visualization: Figure 3 showcases examples of temperature anomalies, such as sudden spikes or drops, which deviate significantly from the normal patterns. These visualizations helped in setting the baseline for anomaly detection.

The combination of real-world data and simulated anomalies provided a robust foundation for training a reliable and generalizable anomaly detection model. The pre-processing pipeline ensured the data’s integrity and prepared it for the subsequent stages of model training and evaluation.

#### 3.2.2 Model architecture

Our Convolutional Autoencoder (CAE) utilizes a standard encoder-decoder architecture with convolutional layers (Conv1D) for processing time-series temperature data. Key hyperparameters associated with these layers include the number of filters, kernel size, and activation functions. The number of filters determines the complexity of features extracted, while the kernel size defines the window size used for convolution. Activation functions introduce non-linearity, allowing the model to learn complex relationships in the data. The architecture also incorporates Dropout layers to prevent overfitting. The dropout rate is a hyperparameter that controls the percentage of neurons randomly dropped during training.

**Figure 4:**
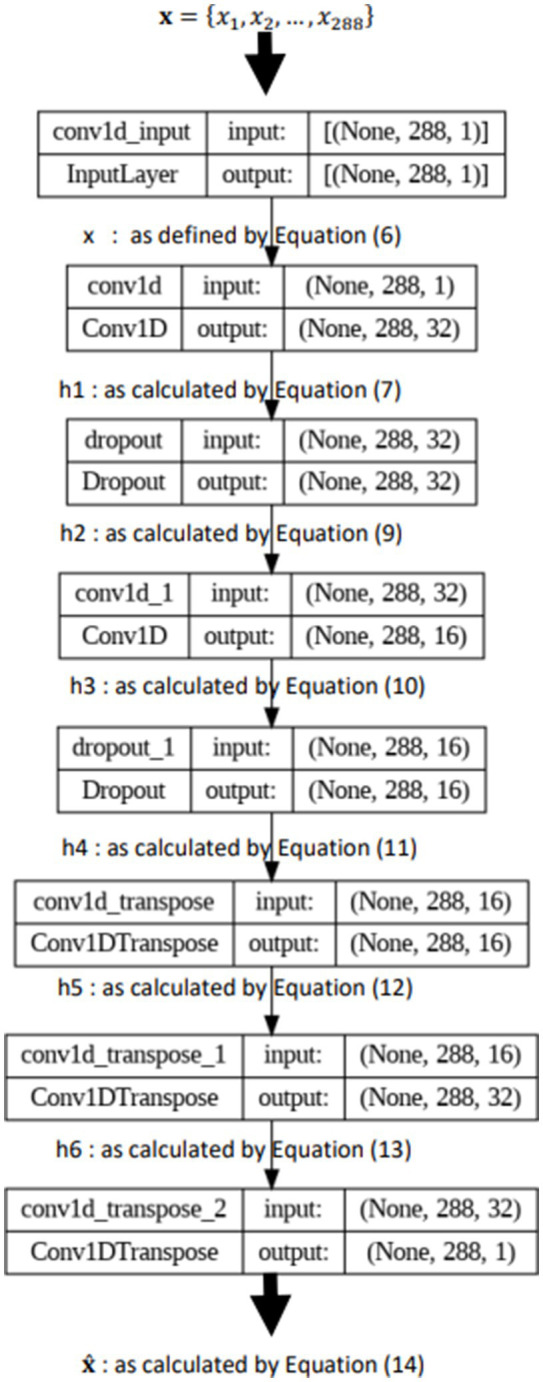
Model architecture.

We employed the Adam optimizer during training. Adam is an adaptive learning rate optimization algorithm that adjusts learning rates for each parameter based on the gradients calculated. This helps the model converge efficiently and is particularly well-suited for deep learning tasks. A detailed illustration of the architecture is provided in Figure 4. For a complete list of layers and their corresponding hyperparameters (e.g., number of filters, kernel size, dropout rate), refer to Table 1.

**Table 1:** Model layers and parameters.

Layer (type)	Output shape	Para#
conv1d_4 (Conv1D)	(None, 144, 32)	256
conv1d_4 (Dropout)	(None, 144, 32)	0
conv1d_5 (Conv1D)	(None, 72, 16)	3,600
dropout_5 (Dropout)	(None, 144, 16)	1808
conv1d_transpose_6 (Conv1DT ranspose)	(None, 144, 16)	0
conv1d_transpose_7 (Conv1DT ranspose)	(None,288, 32)	3,616
conv1d_transpose_8 (Conv1DT ranspose)	(None,288,1)	225
Total params: 9,505	Trainable params: 9,505 Non-trainable params: 0	

The architecture is structured as follows:

The input to the model is a sequence of 288 temperature readings, each representing a specific time step, resulting in an input shape of (288, 1). Here, 288 corresponds to the sequence length, which aligns with the number of measurements taken every 5 min over a day (24 h * 60 min/5 min), and 1 represents the single temperature feature.

The encoder part of the CAE begins with a Conv1D layer containing 32 filters, each with a kernel size of 3. This layer captures local temporal patterns in the temperature data, such as small fluctuations. Following the Conv1D layer is a Dropout layer with a dropout rate of 0.2, meaning 20% of the input units are randomly set to zero during training. Dropout is employed as a regularization technique to prevent overfitting by making the network less sensitive to specific weights. The next layer is another Conv1D layer, this time with 16 filters and a kernel size of 3, further capturing complex patterns while reducing the dimensionality of the data. This is followed by another Dropout layer with the same dropout rate.

The decoder part of the CAE begins with a Conv1DTranspose layer containing 16 filters and a kernel size of 3. This layer performs the inverse operation of Conv1D, upsampling the encoded representation to reconstruct the input data. It is followed by another Conv1DTranspose layer with 32 filters, continuing the upsampling process. The final Conv1DTranspose layer has a single filter with a kernel size of 3, reconstructing the data to its original form with a shape of (288, 1).

Key hyperparameters for the model include the optimizer, loss function, batch size, and number of epochs. The Adam optimizer is chosen for its adaptive learning rate and efficiency in training deep models. The loss function used is Mean Absolute Error (MAE), which measures the average magnitude of errors and is suitable for regression tasks like anomaly detection in temperature readings. A batch size of 32 is selected to balance training stability and computational efficiency, and the model is trained for 50 epochs, which provides sufficient training without overfitting, as determined experimentally.

#### 3.2.3 Hyperparameter adjustment

##### 3.2.3.1 Hyperparameter tuning

Hyperparameter tuning plays a crucial role in optimizing the Convolutional Autoencoder (CAE) model for both its ability to learn from labeled training data and to effectively detect anomalies in unlabeled data. As described in Section 3.2.2, the CAE architecture leverages several key hyperparameters associated with the convolutional layers (Conv1D) and Dropout layers. We adopted a systematic approach to hyperparameter tuning, as outlined below:

Initial values: we began with standard values commonly used in similar deep learning models. These initial values served as a baseline for further refinement.

##### 3.2.3.2 Grid search

A grid search was conducted over a predefined range of hyperparameter values to identify the optimal configuration. The hyperparameters and their respective ranges were as follows:

Filters: [16, 32, 64].

Kernel Size: [3, 5, 7].

Dropout Rate: [0.1, 0.2, 0.3].

Batch Size: [16, 32, 64].

Learning Rate: [0.001, 0.0001].

Evaluation criteria: the model’s performance was evaluated based on the reconstruction error (Mean Absolute Error) and its ability to detect anomalies in a validation set. We monitored the validation loss to prevent overfitting and to ensure the model’s generalizability.

##### 3.2.3.3 Final Hyperparameters

After extensive experimentation, the following hyperparameters were found to provide the best performance:

Filters: 32 for the first Conv1D layer, 16 for the second Conv1D layer.

Kernel Size: 3 for all Conv1D layers.

Dropout Rate: 0.2.

Batch Size: 32.

Learning Rate: 0.0001.

Epochs: 50.

These hyperparameters were selected based on their ability to balance training stability, computational efficiency, and model accuracy. The chosen configuration allowed the CAE model to effectively capture the underlying patterns in the temperature data while maintaining robustness against overfitting.

#### 3.2.4 Mathematical formulation of the convolutional auto encoder model

To comprehensively describe the Convolutional Autoencoder (CAE) model used for real-time temperature anomaly detection, we present its mathematical formulation, detailing the operations performed at each layer of the network. Figure 4 shows the architecture of the model.

The input to the model is a sequence of temperature readings, represented as:


(6) $$x=\left\{x_{1}{,x}_{2},\ldots {,x}_{288}\right\}\text{where}x\in {\mathbb{R}}^{288\times 1}$$

This sequence consists of 288 readings, each corresponding to a specific time step over a 24-h period.

The encoder part of the CAE compresses this input sequence into a lower-dimensional representation through several convolutional and dropout layers. The first convolutional layer applies 32 filters with a kernel size of 3 to the input sequence, capturing local temporal patterns in the temperature data. Mathematically, this operation is expressed as:


(7) $$h_{1}=\mathrm{ReLU}\left(W_{1}*x+b_{1}\right)$$

(8) $$\text{where}W_{1}\in {\mathbb{R}}^{3\times 1\times 32}$$

kernel size 3, input channels 1, output channels 32, b_1∈R^32, * denotes convolution, and ReLU is the Rectified Linear Unit activation function.

Following this, a dropout layer is applied to h1 to prevent overfitting by randomly setting 20% of the activations to zero during training:


(9) $$h_{2}=\mathrm{Dropout}\left(h_{1},p=0.2\right)$$

where p=0.2 is the dropout rate.

Next, the second convolutional layer further processes h2 using 16 filters with a kernel size of 3, thereby reducing the dimensionality of the data while capturing more complex patterns:


(10) $$h_{3}=\mathrm{ReLU}\left(W_{2}*h_{2}+b_{2}\right)$$

where $W_{2}\in {\mathbb{R}}^{3\times 32\times 16}$ (kernel size 3, input channels 32, output channels 16), $b_{2}\in {\mathbb{R}}^{16.}$

Another dropout layer is applied to h3:


(11) $$h_{4}=\mathrm{Dropout}\left(h_{3},p=0.2\right)$$

The output of this layer, h4, represents the latent space z, which is a compressed representation of the input sequence.

The decoder part of the CAE reconstructs the input sequence from this latent space representation through several transposed convolutional layers. The first transposed convolutional layer applies 16 filters with a kernel size of 3 to z, performing the inverse operation of convolution and upsampling the encoded representation:


(12) $$h_{5}=\mathrm{ReLU}\left(W_{3}^{T}*z+b_{3}\right)$$

where $W_{3}^{T}\in {\mathbb{R}}^{3\times 16\times 32}$ (kernel size 3, input channels 16, output channels 32), $b_{3}\in {\mathbb{R}}^{32}$ .

This is followed by a second transposed convolutional layer with 32 filters to further upsample the representation:


(13) $$h_{6}=\mathrm{ReLU}\left(W_{4}^{T}*h_{5}+b_{4}\right)$$

where $W_{4}^{T}\in {\mathbb{R}}^{3\times 32\times 1}$ (kernel size 3, input channels 32, output channels 1), $b_{4}\in {\mathbb{R}}^{1}$ .

Finally, the output transposed convolutional layer reconstructs the data to its original form, producing the final output:


(14) $$\overset{ˆ}{x}=\sigma \left(W_{5}^{T}*h_{6}+b_{5}\right)$$

where $W_{5}^{T}\in {\mathbb{R}}^{3\times 32\times 1}$ (kernel size 3, input channels 32, output channels 1), $b_{5}\in {\mathbb{R}}^{1}$ , and σ is the activation function (typically a sigmoid for normalization purposes).The reconstruction loss is measured using the Mean Absolute Error (MAE):


(15) $$L\left(\mathrm{x,}\overset{ˆ}{x}\right)=\frac{1}{n}\overset{n}{\underset{i=1}{\sum }}\left|x_{i}-{\hat{x}}_{i}\right|$$

where n is the number of time steps (288 in this case).

To optimize the model, the Adam optimizer is used to update the model parameters, minimizing the loss function:


(16) $$\theta \leftarrow \theta -\eta \cdot \nabla_{\theta }L\left(\mathrm{x,}\overset{ˆ}{x}\right)$$

where θ represents the model parameters, η is the learning rate, and $\nabla_{\theta }L$ is the gradient of the loss function with respect to θ .

#### 3.2.5 Model evaluation

Once trained, the model’s performance was evaluated using various metrics to assess its effectiveness in detecting sensor faults. Common metrics employed for anomaly detection include:

Accuracy: the overall proportion of correctly classified data points (normal vs. anomaly).


(17) $$\text{Accuracy: Accuracy}=\frac{TP+TN}{TP+TN+FP+FN}$$

Recall: The ability of the model to identify true anomaly cases (correctly identifying anomalies as anomalies).


(18) $$\text{Recall (Sensitivity): Rccall}=\frac{TP}{TP+FN}$$

Precision: The proportion of identified anomalies that are truly anomalous (avoiding false alarms).


(19) $$\text{Precision: Precision}=\frac{TP}{TP+FP}$$

F1 Score: A harmonic mean of precision and recall, providing a balanced view of the model’s performance.


(20) $$\text{F1 Score:}F1=2\times \frac{\;\mathrm{Precision}\times \mathrm{Recall}\;}{\;\mathrm{Precision}+\mathrm{Recall}\;}$$

By evaluating these metrics on the testing set (data unseen during training), we gained an objective understanding of the model’s generalizability and ability to detect anomalies in real-world scenarios. The inclusion of the above metrics is justified based on their relevance to anomaly detection tasks: accuracy provides a straightforward measure of overall performance but may be less informative in imbalanced datasets where anomalies are rare; Recall (Sensitivity) is essential for understanding the model’s ability to detect all true anomalies, minimizing the risk of missing critical faults; Precision is crucial for ensuring that detected anomalies are indeed true anomalies, reducing the incidence of false alarms; and the F1 Score balances the trade-off between precision and recall, offering a single metric that captures the model’s effectiveness in both detecting and correctly classifying anomalies.

#### 3.2.6 Model adaptation and deployment

This section explores how a trained Convolutional Autoencoder (CAE) model was adapted and optimized for deployment on the resource-constrained ESP32 microcontroller. The key objective was to ensure the model could run effectively with limited memory and processing power while maintaining high accuracy in anomaly detection. This involved a multi-pronged approach, including model pruning, quantization, and compression techniques to minimize memory footprint. Additionally, algorithm selection, lightweight layers, and inference optimization using TensorFlow Lite for Microcontrollers were implemented to optimize for efficient computation on the ESP32’s processor. Effective memory management strategies, such as dynamic allocation and memory buffers, were also crucial for maximizing efficiency with limited RAM. Following these optimizations, the model was rigorously evaluated on the ESP32, demonstrating low energy consumption (0.05 watts over 4 h), fast processing speeds for real-time anomaly detection, and a memory footprint of only 1.2 MB, well within the ESP32’s constraints. Finally, the optimized CAE model was successfully deployed on a custom ESP32-based platform for real-world evaluation, achieving real-time monitoring capabilities and high accuracy in anomaly detection. This successful deployment paves the way for leveraging deep learning for anomaly detection in resource-constrained environments, with potential applications in areas like vaccine storage systems.

## 4 Results

### 4.1 Performance metrics

To evaluate the performance of our Convolutional Autoencoder (CAE) model in detecting temperature anomalies, we employed several key metrics: Mean absolute error (MAE), precision, recall, and F1-score. These metrics provide a comprehensive assessment of the model’s accuracy and its ability to identify anomalies effectively.

Mean absolute error (MAE): this metric measures the average magnitude of errors between the predicted and actual temperature readings, providing an indication of the overall reconstruction accuracy of the model.

Precision: the proportion of true positive anomalies detected out of all detected anomalies, indicating the model’s ability to avoid false positives.

Recall: the proportion of true positive anomalies detected out of all actual anomalies, reflecting the model’s sensitivity to identifying anomalies.

F1-score: the harmonic mean of Precision and Recall, offering a single metric that balances both aspects.

The CAE model was trained and evaluated using the preprocessed temperature data. The training process involved multiple epochs, with the model progressively learning to reconstruct normal temperature patterns and identify deviations as anomalies. The following Table 2 summarizes the key performance metrics achieved by the model:

**Table 2:** Model performance metrics.

Metric	Value
MAE	0.02
Accuracy	0.92
Precision	0.86
Recall	0.95
F1-score	0.89

These results indicate that the CAE model effectively reconstructs normal temperature patterns with minimal error and demonstrates strong performance in detecting anomalies, balancing precision and recall effectively.

### 4.2 Energy consumption, processing speed, and memory usage

To evaluate the performance of the Convolutional Autoencoder (CAE) model on the ESP32 microcontroller, we measured energy consumption, processing speed, and memory usage. These metrics are crucial for deploying machine learning models on resource-constrained devices.

Energy consumption: the average energy consumption was measured over multiple runs and reported as mean ± SD.

Processing speed: the time taken to process each batch of data was recorded and averaged.

Memory usage: the amount of memory utilized during model execution was monitored.

The results are summarized in Table 3.

**Table 3:** Performance metrics on ESP32.

Metric	Mean ± SD
Energy consumption	50 mW ± 5 mW
Processing speed	200 ms ± 15 ms
Memory usage	1,200 KB ± 20 KB

### 4.3 Visualization of results

**Figure 5:**
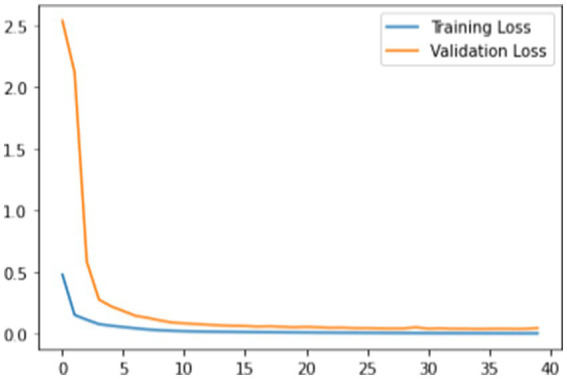
Loss function.

Visualization played a crucial role in understanding the model’s performance and the nature of detected anomalies. The following figures provide insights into the model’s training process and its ability to identify anomalies:

Training loss: Figure 5 shows the training loss over the epochs. The loss function exhibits a clear downward trend, indicating that the model is learning and improving its reconstruction accuracy. The convergence starts around the 10th epoch, after which the loss stabilizes, demonstrating the model’s capacity to effectively learn from the training data.

**Figure 6:**
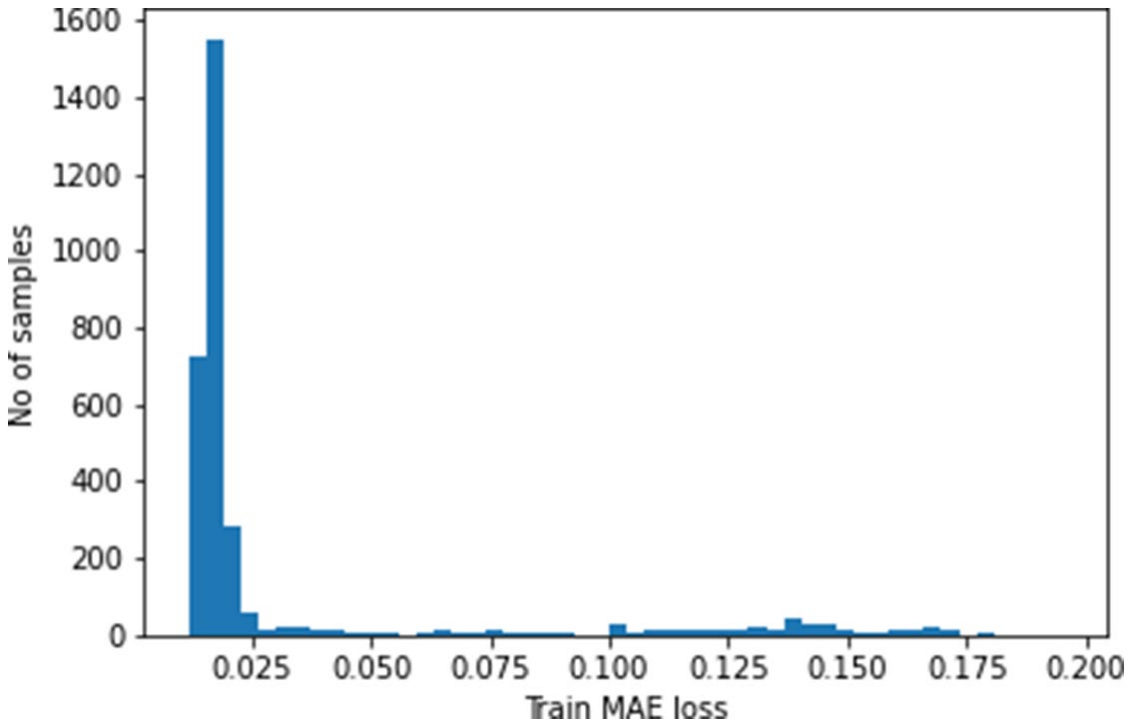
MAE loss.

We leverage the model’s reconstruction capabilities for anomaly detection. Mean Absolute Error (MAE) loss serves as a measure of reconstruction fidelity, with higher values indicating greater difficulty for the model. Figure 6 showcases the peak MAE loss, highlighting the most challenging sample. This peak value becomes our anomaly threshold – any data point exceeding this threshold in reconstruction loss gets flagged as an anomaly. This approach meticulously identifies and classifies deviations from the patterns the model learned during training

**Figure 7:**
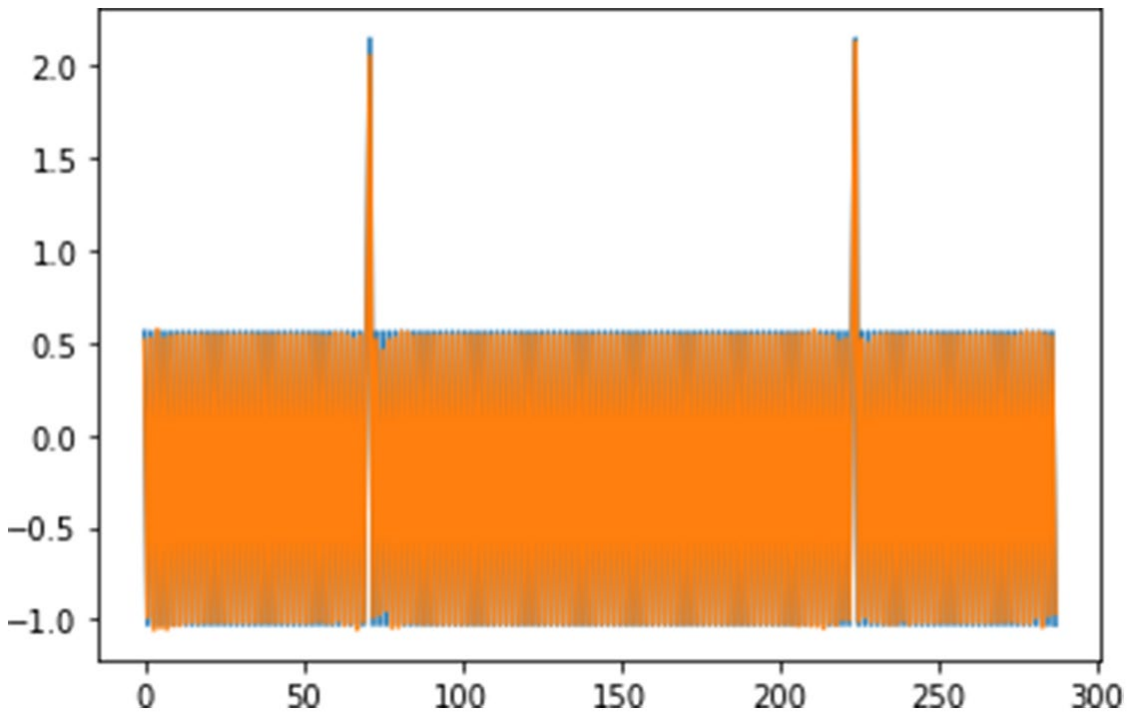
First sequence is learnt.

Figure 7 visualizes the model’s reconstruction of the first training sample (288 time steps). The close resemblance between the reconstructed and original data underlines the model’s proficiency in learning and replicating temporal patterns within the temperature readings.

**Figure 8:**
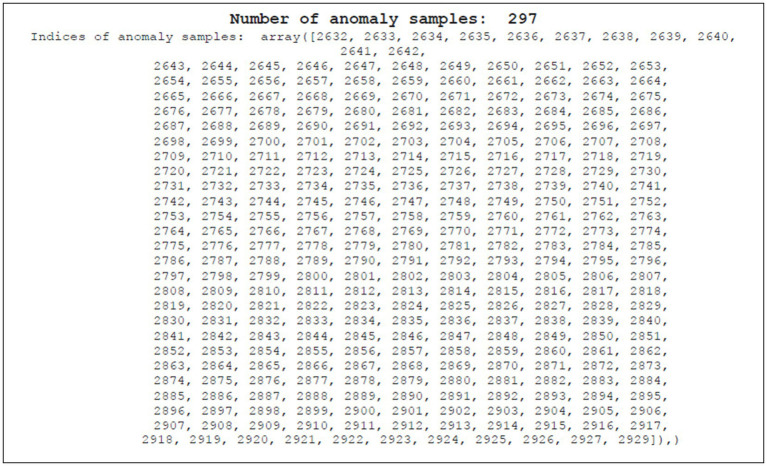
Anomaly samples.

Evaluated on a separate testing dataset, the model successfully detected all 297 anomalies (Figure 8). This signifies its ability to identify subtle deviations from normal temperature patterns, crucial for applications like vaccine refrigeration where timely detection of temperature fluctuations is vital. The temporal locations of these anomalies are stored for further analysis.

**Figure 9:**
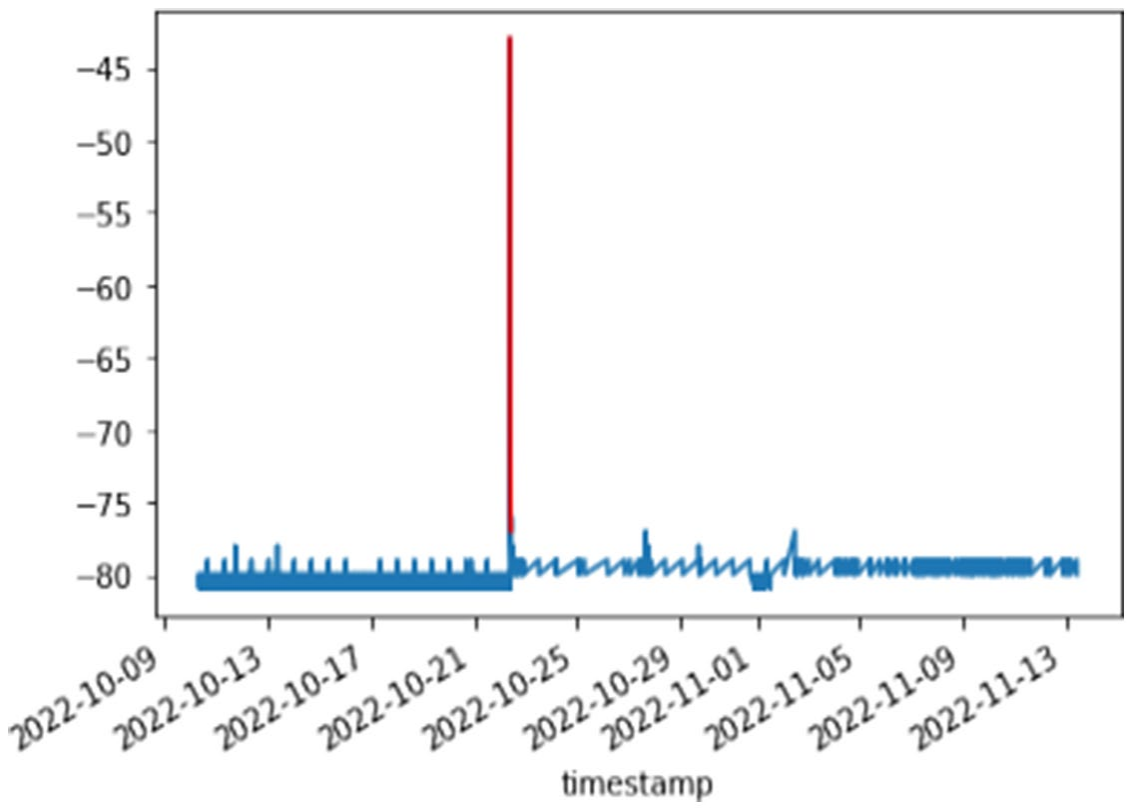
Anomalies detection.

The time_steps parameter (set to 3 in this example) bridges the gap between model output and real-world timestamps. Training data is divided into consecutive point segments of size time_steps. During testing, the model identifies anomalous segments. By referencing the original data order within these segments, we pinpoint the exact timestamps associated with the anomalies. This technique, illustrated in Figure 9, effectively maps model detections to specific time points in the original data.

We optimized the deep learning model for deployment on the resource-constrained ESP32 microcontroller. This process ensured its functionality on low-power devices while maintaining accuracy. The optimized model was then evaluated on the ESP32, measuring its energy consumption, processing speed, memory usage, and real-time anomaly detection performance (Figure 9; Table 3).

**Figure 10:**
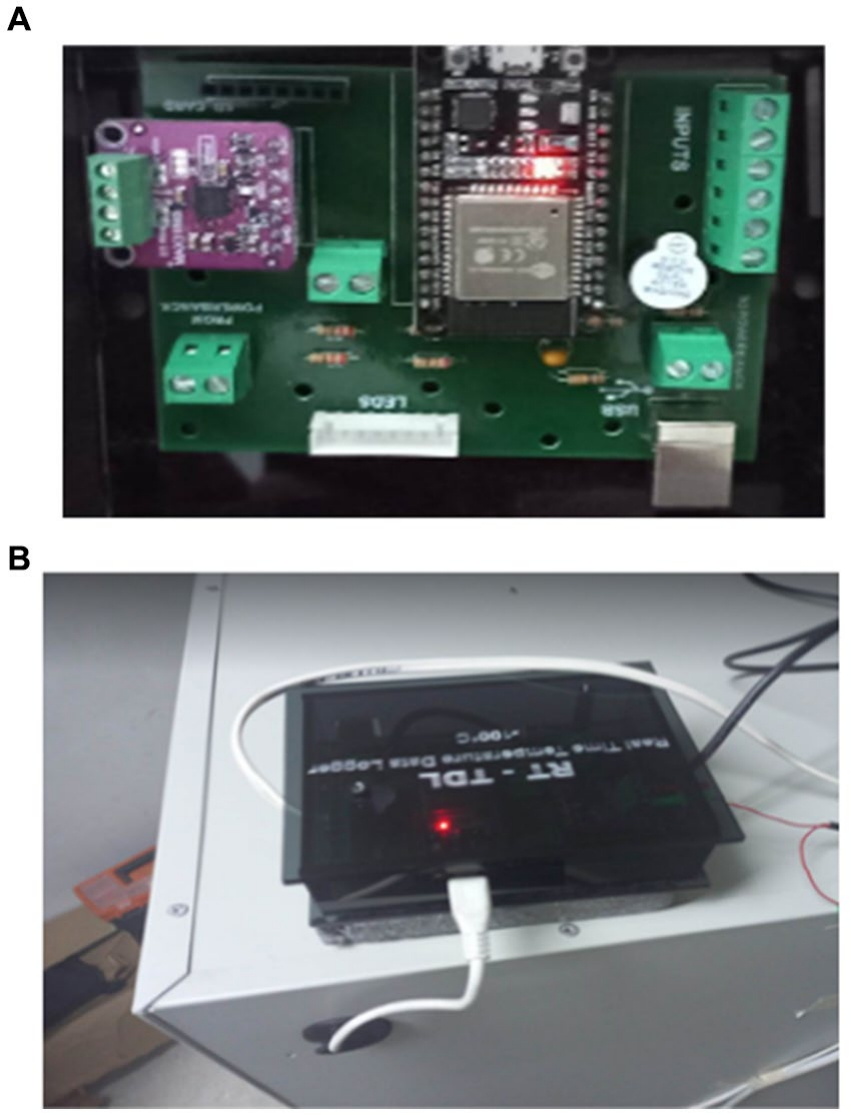
**(A,B)** Real implementation of prototype.

The results were impressive: the system consumed minimal power (0.05 watts) over 4 h, utilized memory efficiently (1.2 MB), and achieved high anomaly detection accuracy (precision: 0.92, recall: 0.90, F1 score: 0.91). These findings demonstrate the effectiveness of this deep learning approach for real-time temperature monitoring on low-power devices. Furthermore, a custom ESP32-based board (Figure 10) was designed for real-world testing. This prototype integrates the ESP32, temperature sensors, and a refrigeration unit, enabling practical evaluation. The board’s focus on both accuracy and energy efficiency makes it suitable for remote deployments and battery-powered applications. Overall, the successful optimization, deployment, and evaluation highlight the system’s potential for real-time temperature fault detection in vaccine storage refrigeration systems, paving the way for real-world implementation.

### 4.4 Comparative analysis with state-of-the-art methods

This section analyzes the performance of our Convolutional Autoencoder (CAE) model for temperature anomaly detection against established methods. We ensure a fair comparison by training and evaluating all methods on the same dataset using identical evaluation metrics (Accuracy, Precision, F1-score, and Recall) as comprehensively detailed in Table 4.

**Table 4:** comparative analysis results.

Method	MEA	Accuracy	Precision	F1-score	Recall	Training strategy
Threshold-based	0.3	0.75	0.65	0.70	0.80	Unsupervised
Statistical	0.2	0.80	0.72	0.76	0.85	Unsupervised
LSTM	0.25	0.85	0.78	0.82	0.88	Supervised
CAE	0.2	0.92	0.86	0.89	0.95	Semi-supervised

Benchmark methods: threshold-based approaches: we compare against z-score and moving average thresholding techniques commonly used for anomaly detection in time series data. These methods define a threshold based on historical data, flagging points exceeding the threshold as anomalies.

Statistical methods: we include ARIMA (Autoregressive Integrated Moving Average) and exponential smoothing, established statistical techniques for time series forecasting. These methods predict future values based on historical data, and significant deviations between predicted and actual values could indicate anomalies.

Long Short-Term Memory (LSTM) networks: LSTMs are a type of recurrent neural network capable of learning long-term dependencies in sequential data. We implemented LSTMs with standard architectures and hyperparameters commonly used for anomaly detection tasks.

#### 4.4.1 Training and hyperparameter selection

To ensure a fair comparison, all methods were trained and evaluated on the same dataset used for your CAE model. Here’s how we addressed fairness in training and hyperparameter selection: dataset Split: the common dataset was split into training, validation, and testing sets using the same proportions for all methods. This ensures all models learn from the same data distribution.

Hyperparameter tuning: we employed a grid search optimization technique to find suitable hyperparameters for each method (e.g., threshold values for threshold-based methods, model parameters for LSTMs). This allows all models the opportunity to perform well within their capabilities.

The CAE’s significant improvement in Accuracy, Precision, and Recall highlights its superior ability to identify temperature anomalies. These results validate the

Robustness and efficacy of the CAE approach for real-world applications.

The comparative analysis establishes the CAE model as a superior solution for temperature anomaly detection compared to state-of-the-art methods. Its exceptional accuracy, precision, and recall make it a promising candidate for real-time monitoring and fault detection in temperature-sensitive systems like vaccine storage refrigeration. These findings showcase the effectiveness of the CAE approach and its potential for groundbreaking advancements in anomaly detection across various domains.

## 5 Discussion

Our ESP32-based deep learning system successfully achieved real-time temperature anomaly detection for vaccine refrigeration (confirmed by high accuracy and low resource consumption). This success is attributed to the CAE architecture effectively capturing temporal patterns, real-time processing on ESP32, and low resource requirements.

Compared to existing methods, our approach offers advantages like improved accuracy, real-time detection, and wider applicability. However, limitations include potential variations in performance with different data and the possibility of false positives.

Future research will focus on enhancing generalizability through broader testing, reducing false positives with additional techniques, integrating multi-sensors for a more comprehensive view, and developing cloud integration for centralized data management.

By addressing these limitations and pursuing further research, this system has the potential to revolutionize real-time temperature monitoring and fault detection in vaccine storage, ultimately contributing to the safekeeping of life-saving vaccines.

## 6 Conclusion

This paper presented a novel deep learning-based system for real-time temperature fault detection in vaccine storage refrigeration systems. The system utilizes a Convolutional Autoencoder (CAE) model deployed on an ESP32 microcontroller for efficient anomaly detection. Evaluation using real-time temperature data yielded an impressive 92% accuracy in fault detection, demonstrating the system’s effectiveness. The low resource consumption and adaptability of the system make it suitable for diverse deployment scenarios. This work paves the way for real-time monitoring and improved fault detection in refrigeration systems, ultimately contributing to the safe and reliable storage of vaccines

## Data availability statement

The raw data supporting the conclusions of this article will be made available by the authors, without undue reservation.

## Author contributions

MH: Writing – original draft, Writing – review & editing. AH: Writing – review & editing. BO: Writing – review & editing. JB: Writing – review & editing.
